# Hybrid Methacrylated Gelatin and Hyaluronic Acid Hydrogel Scaffolds. Preparation and Systematic Characterization for Prospective Tissue Engineering Applications

**DOI:** 10.3390/ijms22136758

**Published:** 2021-06-23

**Authors:** B. Velasco-Rodriguez, T. Diaz-Vidal, L. C. Rosales-Rivera, C. A. García-González, C. Alvarez-Lorenzo, A. Al-Modlej, V. Domínguez-Arca, G. Prieto, S. Barbosa, J. F. A. Soltero Martínez, P. Taboada

**Affiliations:** 1 Department of Chemical Engineering, CUCEI, Universidad de Guadalajara, Guadalajara 44430, Mexico; brendavero.iq@gmail.com (B.V.-R.); taniadzv@hotmail.com (T.D.-V.); carlos.rosales@academicos.udg.mx (L.C.R.-R.); 2 Colloids and Polymers Physics Group, Department of Particle Physics, Faculty of Physics and Health Research Institute of Santiago de Compostela (IDIS), Universidade de Santiago de Compostela, E-15782 Santiago de Compostela, Spain; silvia.barbosa@usc.es; 3 Department of Pharmacology, Pharmacy and Pharmaceutical Technology, I + D Farma Group (GI-1645), Faculty of Pharmacy and Health Research Institute of Santiago de Compostela (IDIS), Universidade de Santiago de Compostela, E-15782 Santiago de Compostela, Spain; carlos.garcia@usc.es (C.A.G.-G.); carmen.alvarez.lorenzo@usc.es (C.A.-L.); 4 Department of Physics and Astronomy, College of Science, King Saud University, Riyadh 11451, Saudi Arabia; amodlej@KSU.EDU.SA; 5 Biophysics and Interfaces Group, Department of Applied Physics, Faculty of Physics, Universidade de Santiago de Compostela, E-15782 Santiago de Compostela, Spain; vicente.domarc@gmail.com (V.D.-A.); xerardo.prieto@usc.es (G.P.)

**Keywords:** gelatin, hyaluronic acid, hydrogel, hybrid scaffolds, tissue engineering, porosity

## Abstract

Hyaluronic acid (HA) and gelatin (Gel) are major components of the extracellular matrix of different tissues, and thus are largely appealing for the construction of hybrid hydrogels to combine the favorable characteristics of each biopolymer, such as the gel adhesiveness of Gel and the better mechanical strength of HA, respectively. However, despite previous studies conducted so far, the relationship between composition and scaffold structure and physico-chemical properties has not been completely and systematically established. In this work, pure and hybrid hydrogels of methacroyl-modified HA (HAMA) and Gel (GelMA) were prepared by UV photopolymerization and an extensive characterization was done to elucidate such correlations. Methacrylation degrees of ca. 40% and 11% for GelMA and HAMA, respectively, were obtained, which allows to improve the hydrogels’ mechanical properties. Hybrid GelMA/HAMA hydrogels were stiffer, with elastic modulus up to ca. 30 kPa, and porous (up to 91%) compared with pure GelMA ones at similar GelMA concentrations thanks to the interaction between HAMA and GelMA chains in the polymeric matrix. The progressive presence of HAMA gave rise to scaffolds with more disorganized, stiffer, and less porous structures owing to the net increase of mass in the hydrogel compositions. HAMA also made hybrid hydrogels more swellable and resistant to collagenase biodegradation. Hence, the suitable choice of polymeric composition allows to regulate the hydrogels´ physical properties to look for the most optimal characteristics required for the intended tissue engineering application.

## 1. Introduction

Tissue engineering (TE) has emerged in the last two decades to address the scarce availability of donors for patients who require a new organ or tissue after failure caused by a disease or trauma [1]. These “synthetic” alternatives can be engineered from a wide combination of different materials able to provide scaffolds to support cell adhesion and a suitable microenvironment for cell proliferation, organization, and subsequent tissue regeneration [2,3,4,5].

Among the different types of materials to construct scaffolds for TE, hydrogels play a key role. These are three-dimensional cross-linked networks of polymers, proteins, and/or peptides [6] able to absorb and maintain large quantities of water inside without being dissolved thanks to their numerous hydrophilic groups, which allow extensive inner chemical and/or physical bonding. Hydrogel characteristics can be tuned by changes in the production manufacturing, composition, cross-linking degree, and microstructure, among others, to control the hydrogel mechanical and structural properties, water retention ability, and cytocompatibility, ultimately aiming to mimic those found in the extracellular matrix (ECM) of many human tissues. Hence, hydrogels provide space, mechanical stability, and a suitable microenvironment in terms of transport of nutrients and biochemical cascade signaling for cell/tissue formation and/or repair [7]. Particularly, the composition and mechanical properties of synthetized hydrogels greatly influence biological cell responses in vitro and in vivo because cells sense the nano/microtopography and the biochemical anchoring points or receptors present in the formed bio-polymeric matrices; besides, adhered cells are influenced by the rigidity/softness of the scaffold, modulating their motility, proliferation, differentiation, and apoptosis responses accordingly [8,9,10].

Proteins such as collagen, elastin, fibronectin, or polysaccharides as hyaluronic acid (HA) are the main components of human ECM [8,11], and thus become excellent biomaterials to construct synthetic biocompatible and biodegradable 3D polymeric scaffolds mimicking the ECM. Gelatin (Gel) is a form of denatured and partially hydrolyzed collagen [12] and a major component of the ECM of several tissues such as cartilage, bone, skin, ligaments, tendon, heart, blood vessels, cornea, and epithelium [13]. When Gel is incubated at temperatures below 23 °C, strong intramolecular hydrogen bonding occurs, turning the gelatin structure into a water-insoluble biocompatible and biodegradable gel free from potential immunological adverse responses. Gelatin bears protease cleavage sites and cell-interactive functional groups, in particular the Arg-Gly-Asp sequence present in adhesion proteins of the natural ECM as fibronectin [1,14], which improves cell adhesion through specific binding with cell membrane αvβ3 integrins. Additionally, Gel-based scaffolds can be easily degraded by metalloproteinases [12], which is essential to allow the deposition of newly formed ECM by cells. However, the main limitation of Gel-based hydrogels for scaffolding relies on their poor thermal and mechanical strength, which are key properties to configure suitable and stable ECM biomimetic scaffolds [15].

Gelatin can be easily cross-linked by methacrylation of the lysine residues of the protein structure upon exposure to UV light to give gelatin-methacryloyl (GelMA) [16]. Methacrylation of Gel helps to form a thermal resistant and rigid 3D polymeric scaffold structure at body temperature (37 °C) with improved physical properties, including degradation, stiffness, and pore architecture [17], and favoring scaffold bioactivity, adhesiveness, and cell spreading properties [18]. For example, GelMA-based scaffolds have been successfully used to develop heart valve-like culture models and scaffolds [19], to enhance endothelial microvascularization [20], to form microvascular channels [18], and to accelerate endochondral bone formation [21], among other TE applications.

However, GelMA is still a relatively weak material that quickly degrades, even in the absence of cells, thus precluding their use in long-time experiments/applications. Therefore, a valid strategy to afford this challenge is to synthetize GelMA hydrogels enriched with other ECM components, such as HA, in order to tune the scaffolds´ properties and control cellular responses [22]. HA, a linear polysaccharide composed of repetitive units of β-1,4-D-glucuronic acid and β-1,3-N-acetyl-D-glucosamine linked by glycosidic bonds [23], is one of the main components of ECM in the central nervous system; cartilage; synovial and vitreous fluids; and connective, cardiac, and epithelial tissues [24]. HA is mainly responsible for wound healing, as well as regulating tissue formation, inflammation, and morphogenesis [25], thanks to its high affinity to adhesion receptors such as cluster of differentiation marker 44 (CD44), intracellular adhesion molecule-1 (ICAM-1), and receptor for HA-mediated motility (RHAMM) [26,27]. Nevertheless, the applicability of HA-based hydrogels is limited by their poor cell adhesiveness, which hinders cell proliferation [17]. As for Gel, the carboxyl and hydroxyl groups of HA can also be chemically modified by methacrylation to yield the formation of cross-linked methacrylated HA (HAMA) hydrogels upon UV light irradiation, which improves their chemical and mechanical properties, providing larger rigidities, high levels of viscoelasticity after swelling [28], resistance to enzymatic degradation compared with unmodified HA [22], and biocompatibility preservation [29]. For example, HAMA hydrogels were proved to stimulate the generation of elastin in valvular interstitial cells, which might be a significant advantage for the fabrication of artificial heart valves [17].

Hybrid hydrogels composed of GelMA and HAMA have been shown to possess outstanding structural and physico-chemical properties. For example, Camci-Unal et al. [12] obtained several hybrid GelMA/HAMA hydrogels and observed that an increase in the total polymer concentration enhances the hydrogel stiffness, which in turn influences the degradation and compressive moduli. Cell spreading in model 2D cell cultures was favored as the hydrogel stiffness increased, whereas the cell spreading capacity in 3D cultures correlated with larger pore sizes and lower stiffness. In another work, Hjortnaes et al. studied the valvular interstitial cell phenotype differentiation on hybrid GelMA/HAMA hydrogels. Softer and less cross-linked hydrogels promoted spontaneous myofibroblast-like differentiation of quiescent fibroblasts, while stiffer ones required the administration of the differentiation factor TGFβ1 to achieve similar cell differentiation levels [30]. In addition, Kuo et al. synthesized hybrid GelMA/HAMA microgels to control the differentiation of induced pluripotent stem (iPS) cells into neurite-like ones. The highest microhydrogel concentrations (high ratios of GelMA) improved cell encapsulation, whereas the lowest ones (high ratios of HAMA) increased the swelling degree and, therefore, hindered cell seeding and spreading [31].

Despite all previous studies, the relationship between structural, physico-chemical, and mechanical properties of GelMA/HAMA hydrogels has not been completely and systematically established. Hence, in the present work, the swelling degree, degradation, pore size, porosity, chemical structure, and rheological properties of hybrid GelMA/HAMA hydrogels were deeply analyzed to correlate the hybrid hydrogel manufacturing and composition with the resulting scaffold structure and physico-chemical properties. An important emphasis was given to their mechanical and rheological characteristics, as it is well known that cells can sense the local stiffness of the ECM and respond by altering integrin expression, focal adhesions, and cytoskeletal organization [32].

## 2. Results and Discussion

### 2.1. Synthesis and Preparation of HAMA-GelMA Hydrogels

**Scheme A1:**
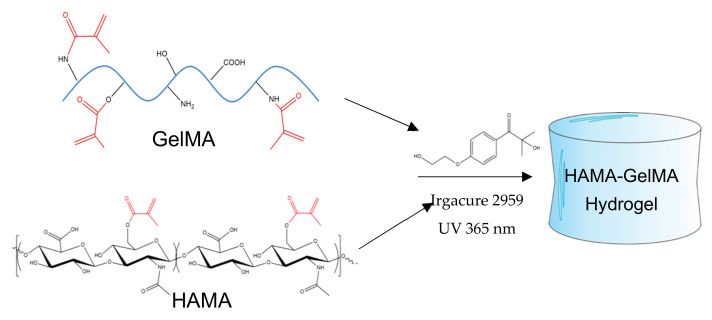
Preparation of hybrid GelMA/HAMA hydrogels.

The chemical properties and composition of unmodified Gel, HA, GelMA, and HAMA hydrogels were characterized. The methacrylation process involved the addition of a methacryloyl group to the amine and hydroxyl residues of Gel and HA (see Scheme A1 in Appendix A) [33,34].

**Figure 1:**
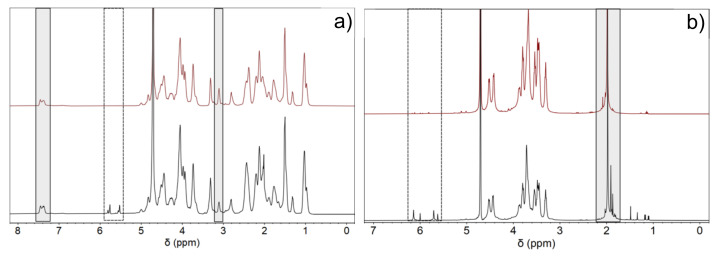
NMR spectra of (**a**) Gel (—) and GelMA (—) and (b) HA (—) and HAMA (—). In (**a**) and (**b**), the dotted rectangles denote the presence of the methacryloyl peaks after successful modification. In (**a**), the grey rectangle at ca. 7.1–7.5 ppm denotes the phenylalanine peaks taken as reference, whereas that one drawn at 3.0–3.3 ppm corresponds to the decrease of lysine groups. In (**b**), the grey rectangle indicates the methyl groups of HA at ca. 2.0 ppm taken as reference and the appearance of methyl protons of methacrylate at ca. 1.9 ppm, respectively.

The degree of methacrylation (DoM) is an important parameter of the crosslinking density of the hydrogel matrix and can largely influence the mechanical properties, structure, and porosity, as well as swelling and degradation abilities [14]. Hence, a high DoM of GelMA produces stiffer hydrogels, which have been shown to favor cell adhesion for prospective TE applications [35]. Here, the amount of photo-initiator used for photopolymerization was adjusted to the number of double bonds in Gel and HA polymeric chains. ^1^H RNM spectra confirmed the successful methacrylation of both biopolymers. Methacryloyl peaks were observed at 5.5 and 5.8 ppm and 6.0 and 6.3 ppm for GelMA and HAMA, respectively, which correspond to acrylate protons (CH_2_ = CH(CH_3_)) of lysine and hydroxylysine (dotted rectangles in Figure 1a,b). A peak at ~1.9 ppm corresponded to methyl protons (CH_2_=CH(CH_3_)) of the grafted methacryloyl group, which was absent in the spectra of both unmodified Gel and HA.

From NMR spectra, the calculated DoM was 40% for GelMA and 11% for HAMA, respectively. Considering the higher DoM of Gel, pure and hybrid hydrogel scaffolds containing this biopolymer as the main component were more stable and stiffer as compared with pure HAMA.

### 2.2. Physico-Chemical Characterization

Pure HAMA, pure GelMA, and hybrid GelMA/HAMA hydrogels were synthetized by photo-crosslinking using Irgacure 2959 as photoinitiator in PBS solutions, with macromers concentrations ranging from 1 to 5% (*w/v*) HAMA and 2 to 10% (*w/v*) GelMA, as reported in Table 1. The gel contents of the scaffolds lie in the 70–100% range. For pure GelMA scaffolds, the gel fraction slightly increases with the increase in protein concentration, while for pure HAMA ones, the gelification extent was 100% irrespective of the polymer concentration. The hybrid GelMA/HAMA hydrogels showed similar behavior to pure GelMA ones, that is, the hydrogel formation is favored as the GelMA concentration in the composition increases, which was further enhanced as HAMA is also added, probably as a consequence of physical bonding, resulting mainly from interchain interactions of electrostatic character between protonated carboxylic groups of HAMA and free lysine ones of GelMA [36]; however, hydrogen bonding between aldehyde, carboxyl, and amine moieties of both polymeric chains and hydrophobic interactions might also play a certain role. These physical interactions would add up to the chemical crosslinks provided by UV-photopolymerization. Non-crosslinked Gel and HA scaffolds dissolved when immersed in deionized water, thus confirming the absence of any chemically crosslinked polymeric network.

**Figure 2:**
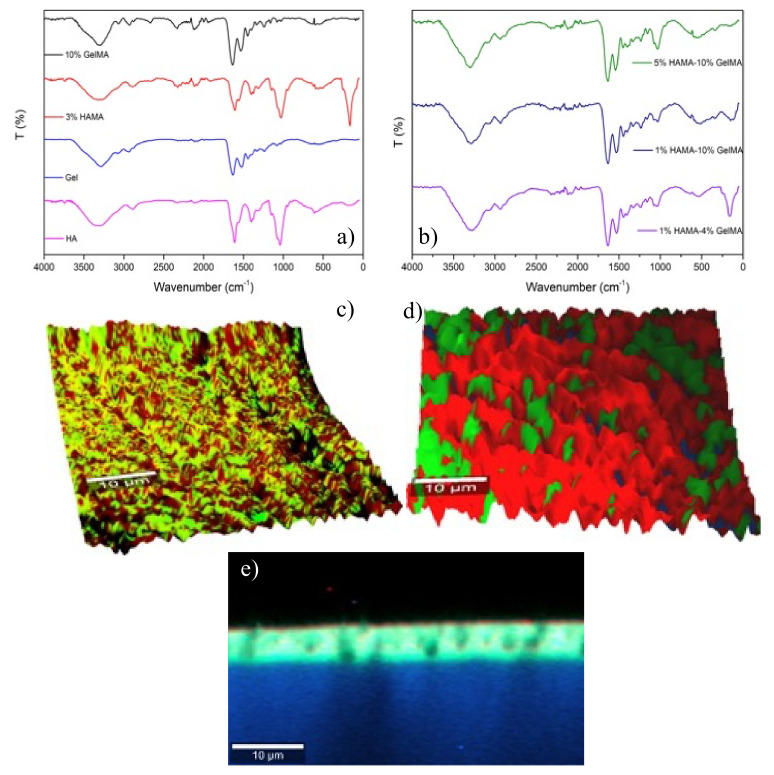
(**a**,**b**) FTIR spectra of selected pure HAMA, GelMA, and hybrid GelMA/HAMA hydrogels at different compositions. Surface topographical composition acquired by Raman confocal 3D imaging of (**c**) 2% GelMA (green)-1% HAMA (red) and (**d**) 5% GelMA (red)-1% HAMA (green) hybrid hydrogels. HAMA was detected using bands at ca. 1380 and 1410 cm^−1^, whereas GelMA was detected using a band of ca. 1455 cm^−1^. (**e**) In-depth Raman imaging of the 2% GelMA-1% HAMA hydrogel. The bright blue-greenish color on the hydrogel in depth denotes the perfect mixing of HAMA (green) and GelMA (blue).

FTIR analyses were performed to identify the interactions and structural components of the hydrogel polymeric matrices. Figure 2a shows the FTIR spectra for pure HAMA, pure GelMA, and hybrid GelMA/HAMA hydrogels. The spectra of HA and Gel scaffolds were similar to those of commercial macromers (not shown). The spectrum of pure HA hydrogels showed a very broad band at ca. 3640–2980 cm^−1^ with a maximum at ca. 3320 cm^−1^, and was attributed to O-H and N-H stretching vibrations of the functional groups engaged in intra- and intermolecular hydrogen bonding between HA molecules [37]. Bands at 2965 and 2815 cm^−1^ corresponded to symmetric and asymmetric C-H stretching modes, whereas bands at 1615, 1560, and 1312 cm^−1^ were attributed to C=O stretching of amide I, N-H bending vibration of amide II, and N-H and C-N vibrations of amide III, respectively. Bands at ca. 1150, 1080–1035, and 950 cm^−1^ were typical of asymmetric stretching of the C-O-C hemiacetalic bridge, C-O stretching in alcohols, and glycosidic linkages between D-glucuronic acid and N-acetyl-D-glucosamine units, respectively [38,39], and bands at ca. 1405 and 1380 cm^−1^ were assigned to CH_2_ and CH_3_ deformation vibrations, respectively [40]. The successful methacrylation of HA (HAMA) was confirmed by the presence of a small band at ca. 1701 corresponding to new C=C bond formation after methacrylation and the slight widening of the shoulder at 1665 cm^−1^ corresponding to the characteristic C=O ester bond associated with methacrylate groups, respectively. Nonetheless, these bands/shoulders were not very intense, in agreement with the low DoM of HA.

The FTIR spectrum of pure Gel scaffolds showed a band between 3660 and 3120 cm^−1^ corresponding to N-H stretching coupled with hydrogen bonding of amide A, 3075 cm^−1^ due to asymmetric stretching vibration of =C-H and NH_3_^+^ of amide B, and 2965–2855 cm^−1^ attributed to C-H stretching vibrations. Moreover, bands at 1635, 1525, and 1235 cm^−1^ correspond to C=O stretching/hydrogen bonding coupled with COO^−^ of amide I, bending vibration of N-H groups and stretching vibrations of C-N groups of amide II, and in-plane vibrations of C-N and N-H groups of bound amide or vibrations of CH_2_ groups of glycine of amide III, respectively. Bands at ca. 1445–1400 cm^−1^ were assigned to the CH_2_ symmetrical deformation mode, whereas those observed at 1165 and 1030 cm^−1^ corresponded to C-O stretching of carboxylic acid and C-N stretching of amines, respectively. Conversely, in the spectra of GelMA hydrogels, bands attributed to amides A, B, and I and CH stretching vibrations were shifted to 3295, 3070, 1638, 1533, and 2985–2835 cm^−1^, respectively.

Hybrid HAMA/GelMA hydrogels presented bands belonging to both polymeric components, whose intensities, which are common to both of them, depended on the scaffold composition. In this sense, band maxima corresponding to amides A, B, I, and II and CH stretching vibrations are located at similar positions as those of pure GelMA, that is, at 3295, 3070, 1635, and 2995–2830 cm^−1^, respectively, as expected owing to the predominance of GelMA in the composition of selected hydrogels (Figure 2b). In addition, small progressive shifts of bands at 1530 to 1540 cm^−1^, 1239 to 1232 cm^−1^, and 1080 to 1070 cm^−1^, corresponding to the amide II band and in-plane vibrations of C-N and N-H groups of bound amide, vibrations of CH_2_ groups of glycine of amide III, and skeletal stretching, respectively, are observed as the total polymer concentration in the hybrid scaffolds increases, and may indicate the involvement of intermolecular interactions between HAMA and GelMA chains upon hybrid gel formation, as indicated previously above [41]. Importantly, an enhancement of the band at ca. 1030 cm^−1^ corresponding to C-O stretching mode in alcohols of the sugar rings of HA was also clearly observed as the concentration of HAMA in the hybrid hydrogel increases, as well as a very small shoulder at ca. 1710 cm^−1^ corresponding to C=C bonding. Hence, IR spectra are compatible with hydrogel formation, mediated by the free radical polymerization of methacrylate groups induced by UV light, but some other contributions may exist, for example, from hydrogen bonding between hydrogen rich moieties of both polymeric chains as well as from electrostatic interactions between carboxylic groups of HAMA and amine groups of lysines of GelMA.

High-resolution confocal Raman microscopy was used to shed further light on the incorporation and distribution of the different polymers within the hybrid hydrogel scaffolds. Figure 2b,c show the combined maps of the individual HAMA and GelMA polymers in the hybrid hydrogels, which were identified using some specific Raman bands of the biopolymers: 1380 and 1410 cm^−1^ for HAMA and 1455 cm^−1^ for GelMA, respectively. As shown in this figure, the stability and preservation of the two initial components, HAMA and GelMA, were achieved, showing a regular distribution and forming a coherent and homogeneous polymeric matrix, as already predicted by FT-IR data. HAMA chains appear well dispersed in a GelMA matrix, with the latter biopolymer being the most prevalent in the hydrogel compositions analyzed. The good mixing of both types of polymers in the hybrid gel matrix can be considered a consequence of a successful photopolymerization process, but also an additional proof of the existence of physical interactions between the polymers, such as electrostatic, ones which probably play a key role in favoring interchain mixing. Interestingly, no interference was observed and, as a result, the surface topography of the scaffold was clearly resolved when performing a 3D projection of the images, as displayed here. To additionally confirm the mixing between both HAMA and GelMA inside the scaffolds, that is, to neglect that it is only taking place at the scaffold surface, in-depth confocal images (i.e., z-stack) were also acquired. The clear marine blue color observed in Figure 2d, which stems from the mixing of the green and blue ones assigned to HAMA and GelMA spectra in the instrument software, respectively, also corroborates the perfect mixing of both components inside the hydrogel, at least along the section the confocal microscope is able to analyze.

### 2.3. Thermal Characterization

**Figure 3:**
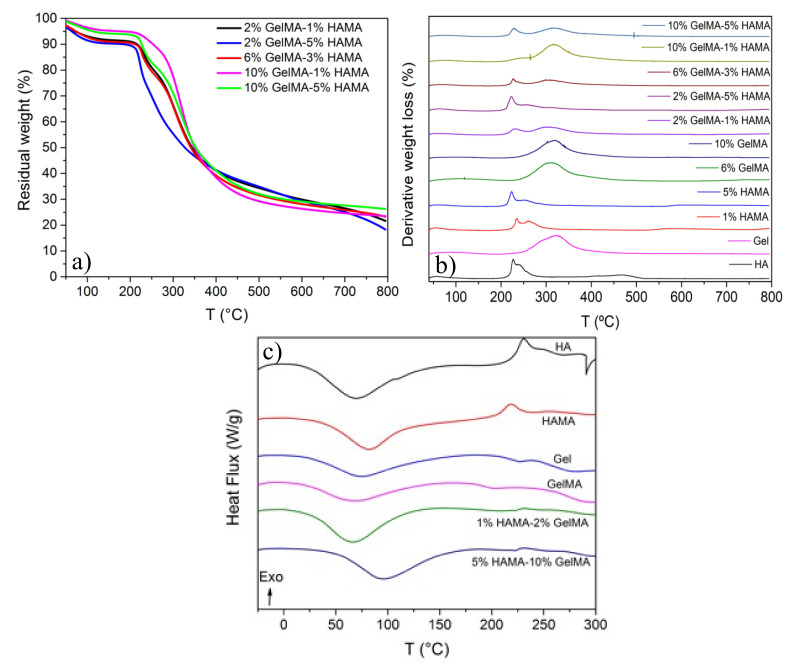
TGA scans of (**a**) hybrid GelMA/HAMA hydrogel scaffolds at different concentrations; (**b**) first derivative plots of TGA data of different pure and hybrid hydrogels; (**c**) examples of DSC curves corresponding pure Gel, pure HA, pure GelMA, pure HAMA, and some hybrid GelMA/HAMA hydrogel scaffolds.

**Figure A1:**
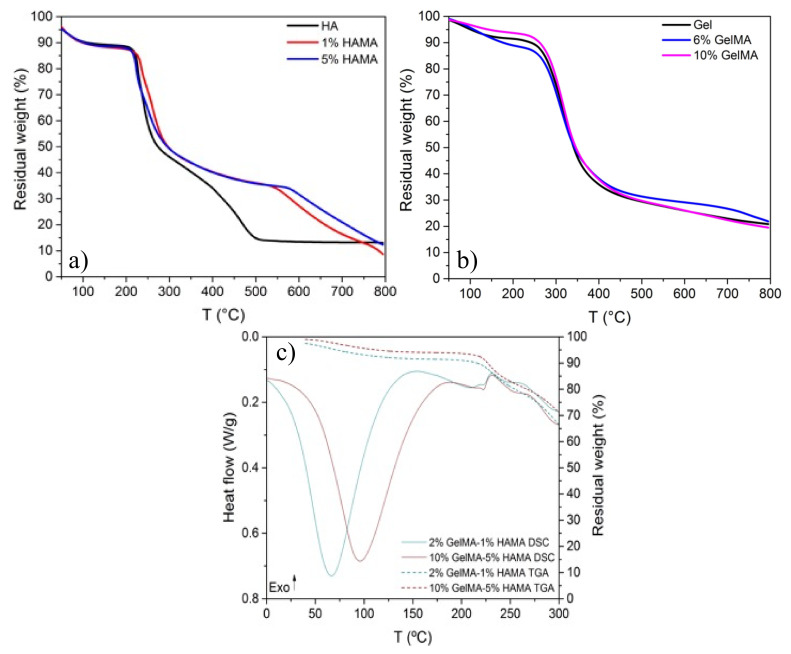
TGA scans of (**a**) pure HA and HAMA and (**b**) pure Gel and GelMA, and (**c**) plots of TGA and DSC runs of hybrid hydrogels with compositions 2% GelMA/1% HAMA and 10% GelMA/5% HAMA for comparison.

TGA curves of pure GelMA, HAMA, and hybrid GelMA/HAMA scaffolds (Figure 3a and Figure A1a,b) indicated that the process of weight loss of the hydrogel scaffolds took place in three main steps. The first one was assigned to the loss of residual water molecules in the samples and occurred in the temperature range of 40–150 °C, where a weight loss of ca. 12% was observed for pure HA and HAMA hydrogels. In the case of pure Gel and GelMA ones, a concentration-dependent behavior was observed, that is, as the protein concentration increased, lower amounts of water were released, i.e., weight losses decreased from 11% to 5% from pure non-crosslinked Gel to 10% GelMA. It is worth mentioning that polysaccharides and proteins have an important affinity for water molecules with different interaction strengths: free water, released at ca. 40–60 °C; water linked through hydrogen bonds, released at 80–120 °C; and water strongly bound through polar interactions to carboxylate groups, released from 160 °C [42]. The second degradation step occurred from 250 to 400 °C for Gel and GelMA (weight losses between 57–63%), and from ca. 200 to 300 °C for HA and 200 to 400 °C for HAMA (with weight losses of 43% and 48%), respectively. Finally, the third one appeared between 400 and 800 °C for Gel and GelMA (weight loss in the range of 9–12%), and between 300 and 500 °C and 500 and 800 °C for HA and HAMA, respectively (weight losses lying of ca. 25–30%).

The degradation of pure Gel scaffolds started at ca. 250 °C with a maximum at 320 °C, T_max_, whereas for pure HA ones, it began at ca. 195 °C with a main maximum at ca. 226 °C and a secondary one at ca. 240 °C. For pure GelMA hydrogels, the degradation slightly shifted to lower temperatures, starting at 243 °C and with a maximum at 312 °C, while for HAMA hydrogels, the degradation began in the range of 187–198 °C, with a first maximum at 224–235 °C and a second one at 250–261 °C, becoming larger as the polymer concentration rises (Figure 3b). Regarding the degradation of the hybrid, scaffolds showed behavior in between pure HAMA and GelMA scaffolds depending on the relative compositions, except at the highest GelMA one. It was observed that the hybrid hydrogels containing larger GelMA concentrations were more resistant to degradation, and the increasing presence of HAMA additionally enhances the thermal stability through the establishment of electrostatic interactions between anionic groups of HAMA and unsubstituted lysine residues of GelMA at least, despite that additional forces such as hydrophobic and/or hydrogen bonding cannot be neglected.

For example, the hybrid hydrogel with the composition of 2% GelMA-5% HAMA showed the most similar thermal degradation behavior to that of pure HAMA ones, as it contained the largest mass of HAMA of all tested hybrid scaffolds; conversely, the hydrogel with the composition of 10% GelMA-1% HAMA showed a similar degradation pattern as that of pure GelMA scaffold. In general, hybrid GelMA/HAMA hydrogels started to be degraded at ca. 190 °C and displayed two main distinctive peaks at ca. 220–225 and 320–325 °C belonging to both HAMA and GelMA, respectively, with the intensity of each peak being proportional to the HAMA and GelMA content in the hybrid scaffold. T_max_ also slightly shifted to higher temperatures with the increasing GelMA concentration in the composition, thus pointing to better thermal stability of the scaffolds, as a consequence of the larger DoM of gelatin, which enhances chemical crosslinking, but also to the larger availability of lysine groups to electrostatically interact with anionic moieties of HA, as mentioned previously.

Pure HA hydrogels degraded quicker, with a degradation extent of ca. 87% at 500 °C, while for HAMA ones, it was only ca. 64%, probably as a consequence of photocrosslinking. Such a difference was not observed for pure Gel and GelMA hydrogels, with degradation extents lying in the range of 67–70% at 500 °C. At 800 °C, pure HA and HAMA hydrogels degraded up to ca. 90–95%, whereas pure Gel and GelMA ones reached values of ca. 80%. Finally, hybrid GelMA/HAMA scaffolds displayed degradation extents close to those observed for pure GelMA ones, lying between 64 and 70% at 500 °C and 72 and 84% at 800 °C, respectively, depending on the composition. Nevertheless, a slightly larger resistance to degradation can be observed for more concentrated hybrid hydrogels.

On the other hand, the DSC data (Figure 3c and Figure A1c) were in good agreement with TGA/DTG experiments. The thermograms showed a broad endothermic peak centered at ca. 71 °C and an exothermic peak at 231 °C for pure HA scaffolds, which were shifted to 82 °C and 218 °C for pure HAMA ones, respectively, as a consequence of photocrosslinking. For pure Gel matrices, two endothermic peaks centered at ca. 74 °C and 281 °C were observed, with a glass transition (T_g_) at 227 °C. These peaks were shifted to 72, 297, and 202 °C, respectively, for GelMA hydrogels. The first endothermic peak was related to the evaporation of residual water linked to HA/HAMA or Gel/GelMA, and its shift to larger temperatures would indicate the lower availability of water and/or that water molecules are more tightly bounded to the polymeric HAMA chains. The exothermic peak for HA and HAMA was attributed to the decomposition of HA chains, and the endothermic peak at ca. 280–300 °C in Gel/GelMA was attributed to the disintegration of the intermolecular side chains of the protein. In the case of hybrid GelMA/HAMA scaffolds, the observed thermal behavior was a combination of that of pure HAMA and GelMA. The first endothermic peak was observed between 66 and 95 °C, and the HAMA exothermic peak slightly shifted to ca. 233 °C. In addition, the Gel T_g_ lying at ca. 210–212 °C was observed, while the second endothermic peak corresponding to the protein shifts above 300 °C was not observed. All these observations are in agreement with a larger resistance to degradation of the hybrid polymeric matrices as a consequence of the good intermixing of the polymeric HAMA and GelMA chains in the scaffold structure, as demonstrated by Raman images.

### 2.4. Rheological Properties of the Scaffolds

**Figure A2:**
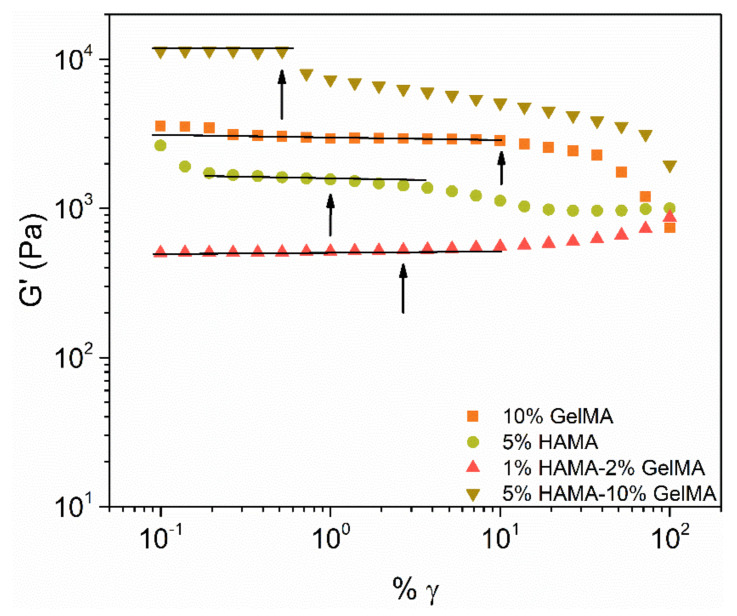
Storage modulus (G’) obtained from oscillatory deformation sweep measured for GelMA, HAMA, and mixtures of HAMA–GelMA at 37 °C.

Figure A2 shows the storage modulus (G′) as a function of deformation for UV cross-linked GelMA, HAMA, and hybrid GelMA-HAMA hydrogels at 37 °C. For 10% GelMA hydrogels, the linear viscoelastic region (LVR) was extended to strain values of ca. 10%, whereas for 5% HAMA hydrogels, the LVR was detected at a deformation of ca. 1%. Similarly, the limit of the linear viscoelastic region of an 10% GelMA-5% HAMA hydrogel appeared at deformations γ < 0.5%; at values from 0.5 to 80%, G′ exhibited a step-like decrease near 0.5%, and then gradually diminished. This decline derived from a gradual breakdown of the gel structure as the applied deformation increased [43].

At strain values higher than 80%, G′ sharply decreased, indicating the collapse of the structure. Additionally, the 10% GelMA-5% HAMA hybrid hydrogel was harder in the linear viscoelastic zone compared with pure 5% HAMA or 10% GelMA alone, as the G′ value of the hydrogels was 3× (1576) and 6× (3024) higher than those of pure HAMA and GelMA hydrogels, respectively. This can be explained by an increase in the crosslinking density in the hybrid hydrogel; however, the LVR reduction indicated that these hydrogels can be easily broken down in comparison with pure HAMA and GelMA ones.

The LVR limit of 2% GelMA-1% HAMA hydrogels increased at deformation values around 3%, whereas G′ in the LVR was lower in more than one order of magnitude, confirming a much lower crosslinking density. Interestingly, 2% GelMA-1% HAMA hydrogels showed a strain hardening above 3% deformation, which is uncommon in synthetic polymeric systems, but has been reported for soft biological hydrogels [44]. This behavior was also detected in fibrin clots for strain amplitudes above 10% wt. and was linked to the formation of networks shaped by highly elongated proteins with mesh sizes in the order of microns [45].

**Figure 4:**
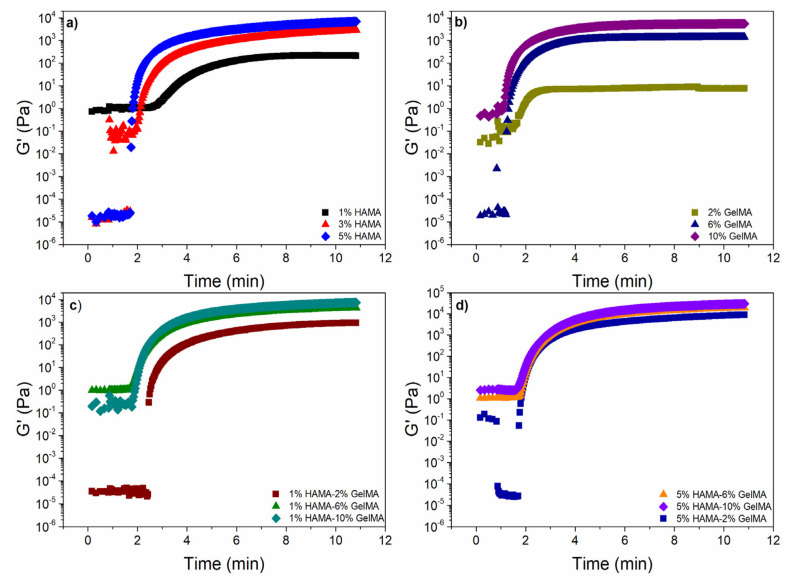
Storage modulus (G′) as a function of the crosslinking time for different concentrations of (**a**) HAMA; (**b**) GelMA; (**c**) 1%HAMA-X% GelMA; and (**d**) 5% HAMA-X% GelMA at 37 °C.

The photo-crosslinking kinetics of all hydrogel samples were studied by oscillatory time sweep experiments, as depicted in Figure 4. At low reaction times for both pure GelMA and HAMA systems, the loss moduli ( G ″) was higher than G′ as a result of the solution state of the hydrogels (sol-state, not shown). Both moduli had a transient plateau up to a time value where G′ increased sharply [46]. This step represented the transition state from a solution (sol-state) to a viscoelastic solid state (gel-state); the onset of this increase was used as the gelation time ($t_{\mathrm{gel}}$). The $t_{\mathrm{gel}}$ diminished with increasing polymer concentrations, as expected, for HAMA or GelMA hydrogels. For hybrid HAMA/GelMA mixtures, $t_{\mathrm{gel}}$ values ranged from 1.6 to 1.8 min, except for the 1% HAMA-2% GelMA, which had similar $t_{\mathrm{gel}}$ values to their alone counterparts (2.49 min). The complete list of $t_{\mathrm{gel}}$ values is shown in Table A1.

Figure 4 also shows G′ values obtained in time sweep experiments for HAMA (Figure 4a), GelMA hydrogels (Figure 4b), and hybrid HAMA/GelMA hydrogels with 1% HAMA-2, 6 and 10% GelMA (Figure 4c), and 5% HAMA-2, 6 and 10% GelMA (Figure 4d).

The plots for all scaffolds exhibited a sharp and rapid increase in G′ followed by a plateau. The G′ values for HAMA hydrogels after 10 min of photoirradiation were very similar to those obtained for GelMA using half the concentration, that is, 1% HAMA (217 Pa), 3% HAMA (2574 Pa), and 5% HAMA (6366 Pa), while for 2% GelMA (7 Pa), 6% GelMA (1382 Pa), and 10% GelMA (5446 Pa), respectively. Hybrid hydrogels with 5% HAMA (2% GelMA, 8255 Pa; 6% GelMA, 18,417 Pa; 10% GelMA, 28,104 Pa) showed G′ values at least one order of magnitude higher than pure HAMA, pure GelMA, and hybrid hydrogels containing 1% HAMA (2% GelMA, 886 Pa; 6% GelMA, 4043 Pa; 10% GelMA, 6868 Pa), as expected from the higher cross-linking density achieved.

**Figure 5:**
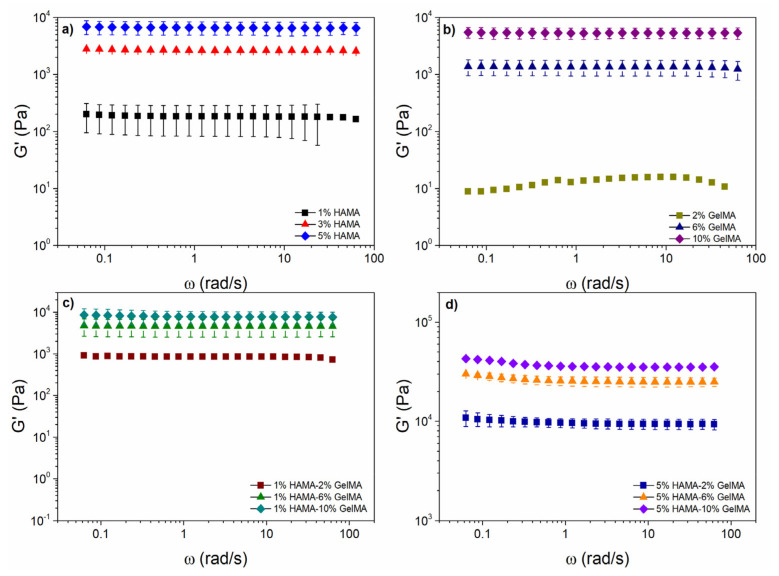
Storage modulus as a function of frequency and (**a**) HAMA concentration; (**b**) GelMA concentration; (**c**) 1%HAMA-X% GelMA; and (**d**) 5% HAMA-X% GelMA. Performed at a deformation within the linear viscoelastic region (LVR) and a temperature of 37 °C.

Figure 5 depicts G′ as a function of frequency for different HAMA (Figure 5a) and GelMA (Figure 5b) concentrations. For pure 1% HAMA and 2% GelMA hydrogels, G′ was nearly frequency-independent, except at values above 20 rad/s. For 1% HAMA (Figure 5a green symbols), the error bars were omitted for the last values owing to the high variation observed at those frequencies. The behavior of HAMA and GelMA hydrogels was like that observed in weak gels of lamellar liquid crystals formed by double tails ionic surfactants, i.e., aerosol OT/water and didodecyldimethylammonium bromide (DDAB) [47,48]. However, for the remaining hydrogels tested, G′ showed a true gel-like behavior with values independent of the frequency.

For 1% HAMA-2% GelMA and 1% HAMA- 6% GelMA samples (Figure 5c), G′ exhibited a true gel-like behavior; however, 1% HAMA-2% GelMA hydrogel showed a slight  G ′ dependence at higher frequencies (>40 rad/s). G′ was much larger (795 Pa) than the one obtained for 2% GelMA (10 Pa) and 1% HAMA (174 Pa), which may be derived from an increase in the crosslinking density. This effect was also observed in the other hybrid hydrogels 1% HAMA- 6% GelMA (4606 Pa) and 1% HAMA- 10% GelMA (7643 Pa), with values higher than 6% GelMA (1306 Pa) and 10% GelMA (5355 Pa).

At frequencies higher than 6.28 rad/s, G′ of 5% HAMA-2% GelMA, 5% HAMA-6% GelMA, and 5% HAMA-10% GelMA hydrogels (Figure 5d) decreased and became independent of the applied frequency. In comparison, the lowest hybrid hydrogel (5% HAMA-2% GelMA) had a higher  G ′ value (9358 Pa) than 1% HAMA-10% GelMA (7643 Pa), which contained a higher weight of added materials (7% vs. 11%) and the highest G′ value observed compared with 5% HAMA (6574 Pa) and 2% GelMA (10 Pa) hydrogels.

**Figure A3:**
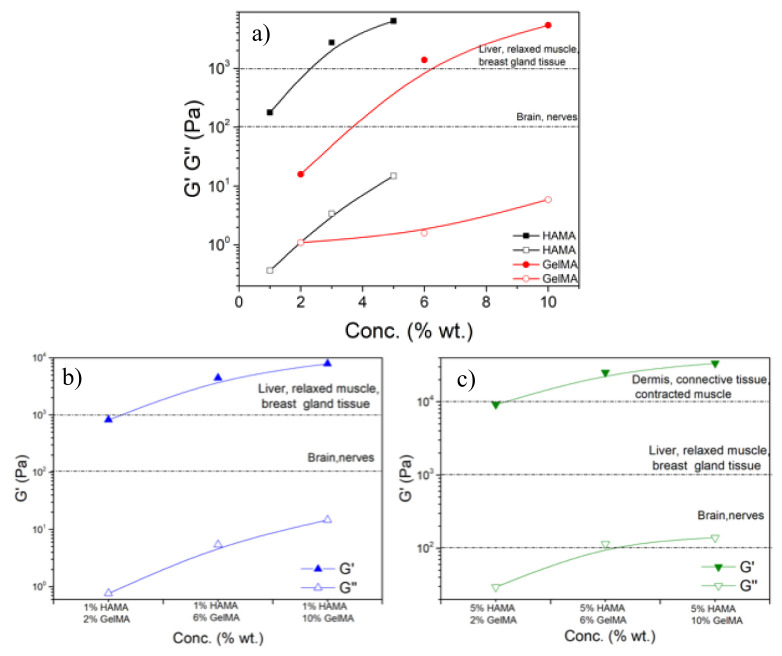
Storage and loss moduli as a function of HAMA and GelMA concentrations for pure (**a**) HAMA and GelMA and (**b**,**c**) hybrid hydrogels at 37 °C and a frequency of 6.28 rad/s. Dashed lines represents the limits of the G′ values for use of the hydrogel as scaffolds for different types of tissues [5]. The continuous lines are to guide the eye.

The variation in the log–log plot for the storage and loss moduli as a function of pure HAMA and GelMA concentrations, at a frequency of 6.28 rad/s, is shown in Figure A3. G′ increased from 2·10^2^ to 6·10^3^ Pa and from 1·10^1^ to 5·10^3^ Pa for HAMA and GelMA hydrogels, respectively.

In this range of hardness, hydrogels can be used as scaffolds for brain tissue, nerves, liver, relaxed muscle, and breast tissue [49]. For HAMA hydrogels,  G ″ increased at a similar rate as G′. This produced harder and stronger gels, as evidenced by the low values of the loss factor (tanδ) [50]. Conversely, for GelMA hydrogels, G′ showed a similar trend to HAMA ones; however, higher concentrations of GelMA were needed to obtain similar G′ values. In addition, the storage modulus values increased at a different rate than the loss modulus ones. The relative increase in G′ over  G ″ caused the gels to become stronger and harder. Nevertheless, the maximum stiffness level achieved for GelMA scaffolds was similar to HAMA hydrogels (Figure A3a).

Figure A3b,c depict G′ and G″ for hybrid HAMA/GELMA hydrogels for 1% HAMA-2,6 and 10% GelMA and 5% HAMA-2,6 and 10% GelMA at 37 °C and 6.28 rad/s, respectively. For the first type of hybrid hydrogel (containing 1% HAMA), both G′ and G″ increased with GelMA concentration at a similar increase rate, which produced harder hydrogels with similar strength values. The storage moduli had values from 8·10^2^ to 9·10^3^ Pa, demonstrating that these hydrogels could be used as scaffolds for liver, relaxed muscle, breast, and gland tissues [51]. For the second type of hybrid hydrogel (with 5% HAMA), both G′ and G″ moduli showed a similar increase rate as that of 1% HAMA hydrogels, but with two main differences: (i) the hardness increased ca. one order of magnitude and the strength was lower, with G′ values from 9·10^3^ to 2·10^4^ Pa; (ii) this hybrid hydrogel can be used as a scaffold for dermis, connective tissue, and contracted muscle tissues [52,53].

**Figure A4:**
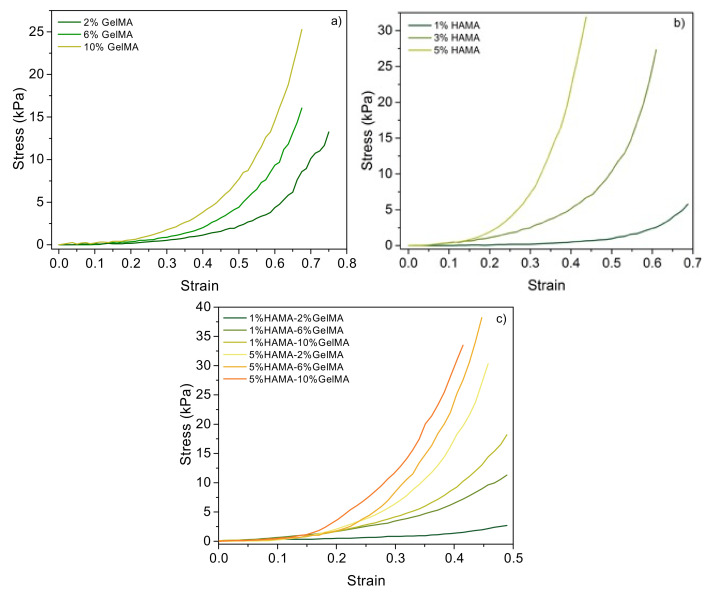
Compression tests for pure (**a**) GelMA and (**b**) HAMA scaffolds and for hybrid (**c**) GelMA–HAMA of different compositions. Concentrations are indicated in the plots.

Data derived from compression tests are shown in Figure A4. The general slope was linear at low strains (<15–20%), and then increased with the strain. Overall, the modulus (i.e., slope of stress versus strain curve at low strain) correlated well with the network cross-linking density (i.e., swelling, see below) and the total polymeric content, being larger as the HAMA concentration in the composition increases. Nevertheless, the values obtained were not very high in agreement with hydrogels showing outstanding swelling abilities.

### 2.5. Structure and Morphology of the Scaffolds

The internal structure and morphology of the scaffolds play a crucial role for their intended use in TE. 

**Figure 6:**
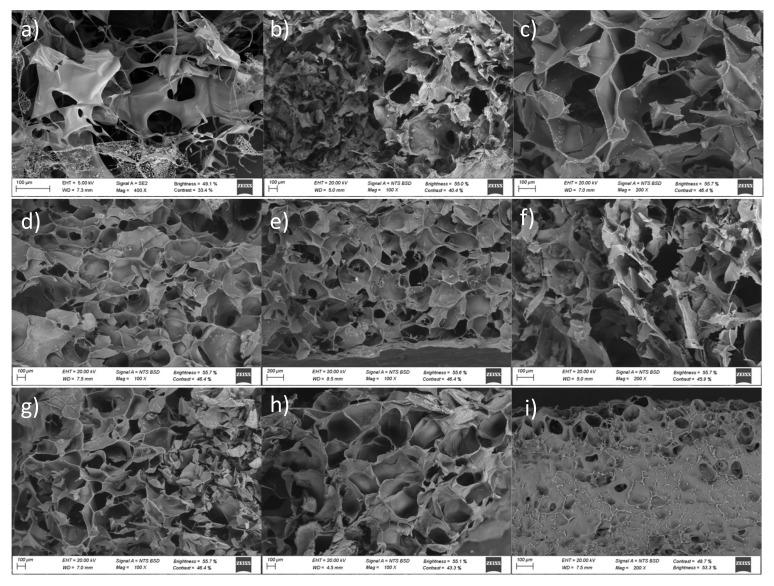
SEM images of (**a**) 1%, (**b**) 3%, and (**c**) 5% HAMA; (**d**) 2% and (**e**) 10% GelMA; (**f**) 1% HAMA-2% GelMA; (**g**) 5% HAMA-2% GelMA; (**h**) 1% HAMA-10% GelMA; and (**i**) 5% HAMA-10% GelMA.

Figure 6 shows FE-SEM images of pure HAMA and GelMA hydrogels as well as hybrid GelMA/HAMA ones at different compositions. Cross-sectional images of pure HAMA hydrogels (Figure 6a–c) showed a non-homogenous, disordered scaffold structure with apparently fragile walls even at the highest polymer concentration (5% *w/v*). The cross-sectional micrographs revealed macropores or voids ranging from ca. 90 to 380 μm depending on polymer concentration (assuming mean equivalent circle diameters). Pure GelMA scaffolds showed an intact and more stable cross-sectional structure (Figure 6d,e) resembling a honeycomb pattern, with more homogeneous pores of smaller sizes ranging from 40 to 250 μm. For both types of pure scaffolds, the hydrogel structure was best defined and pore sizes became smaller as the total hydrogel macromer concentration increased. This behavior may result from the fact that hydrogels with lower polymeric contents may contain more water inside, forming larger ice crystals during freezing, thus giving rise to larger pore sizes after lyophilization [54,55]. These observations were in agreement with the significant differences observed in the hydrogels’ swelling ability, which also decreased as the total polymer content increased, i.e., the porosity was tunable by changing the polymeric concentration [56,57].

Hybrid GelMA/HAMA hydrogels had structural cues and pore sizes between those of both pure HAMA and GelMA scaffolds. Low HAMA concentrations in the hybrid hydrogels (1% *w/v*) slightly enlarged pore sizes regarding those of pure GelMA ones. This would suggest that HAMA chains intermingle with GelMA ones to form a hybridized matrix able to absorb more water and, as a result, the size of ice crystals expanded during freezing, yielding larger voids. Nevertheless, at larger HAMA concentrations, the observed trend was the opposite as a consequence of the larger total polymer contents in hydrogels (Figure 6f–i). An increase in the HAMA weight percentage also gave rise to more rugged void walls, obtaining a scaffold structure more similar to that of pure HAMA ones. This behavior can be a consequence of the lower DoM of HAMA compared with GelMA, which strongly impacts the final hydrogel properties (see below). Higher DoM values have been correlated with more homogeneous and smaller pores inside the polymeric matrix, while enhancing the overall rigidity, as confirmed by rheology. SEM images of hybrid GelMA/HAMA hydrogels also corroborated this point, that is, the structure of hybrid scaffolds was well preserved and pore sizes decreased as the GelMA concentration in the hybrid scaffold increased (Figure 6f–i), ranging, for example, from ca. 75 to 210 μm and 40 to 140 μm for 2% GelMA-1% HAMA and 10% GelMA-1% HAMA scaffolds, respectively. In addition, the distribution of macropore sizes is susceptible to favor vascularization through the smaller macropores and cell colonization and tissue growth through the large ones [58], respectively. This structure should also help in the prevention of fast tissue formation on the external area of the scaffolds that may hinder the access of cells and nutrients towards the interior, thus impeding cell proliferation and structuration inside the scaffolds [59].

SEM observations were complemented with mercury immersion porosimetry (MIP) measurements in order to gain further knowledge about the porous structure of scaffolds. All scaffolds have, in general, good pore interconnections, as confirmed by MIP data (ε_MIP_, open porosities). In particular, relatively high porosities were observed for pure HAMA and GelMA scaffolds, with average pore size values being smaller than those derived from SEM images (Table 1). For hybrid GelMA/HAMA hydrogels, porosity and pore sizes were observed to decrease as the GelMA content in the scaffold increased from 2 to 10% (*w/v*), that is, the volume fraction occupied by the polymeric material itself increased with the protein content, in agreement with density data (Table 1) and SEM images. It is worth mentioning that most of the present hybrid hydrogel compositions had open porosities above 70%, which may be ideal for TE purposes as they should allow a correct permeation of nutrients [60] and facilitate cell colonization and growth [61]. On the other hand, the differences in pore sizes from SEM and MIP data might stem from the limitations of the latter technique related to connectivity and spatial arrangement of pores (i.e., large pores surrounded by smaller pores are only filled with mercury at the pressure needed to fill the latter ones), the upper measurable pore size limit of 180 µm, and the assumptions made in pore size calculations [62].

### 2.6. Swelling Degree

The capacity of hydrogels to swell depends on pore size, cross-linking density, and polymer–polymer and polymer–solvent interactions, and is of key importance for their use in wound healing drug delivery and, of course, TE applications, as it characterizes their ability to absorb body fluids and regulate the transfer of cell nutrients and metabolites [63].

**Figure 7:**
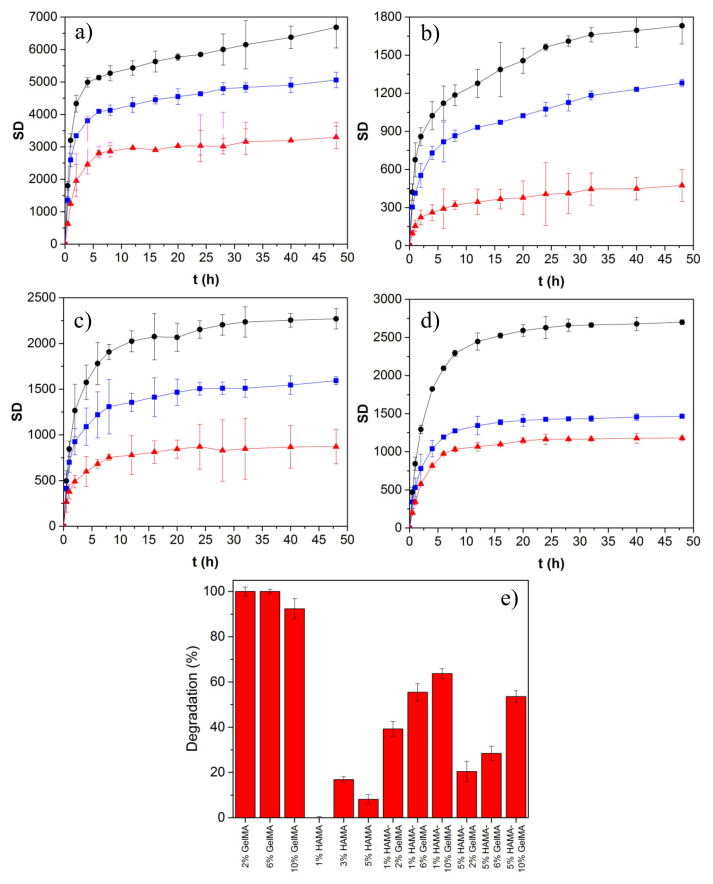
Swelling kinetic data for (**a**) pure HAMA hydrogels of (●) 1, (■) 3, and (▲) 5%; (**b**) pure GelMA ones of (●) 2, (■) 6, and (▲) 10%; and hybrid HAMA-GelMA ones of (**c**) (●) 1–2, (■) 1–6, and (▲) 1–10%; and (**d**) (●) 5–2, (■) 5–6, and (▲) 5–10%, respectively. (**e**) Degradation of scaffolds by enzymatic attack after 24 h of incubation at 37 °C.

Figure 7 shows the time evolution of hydrogel swelling for the different pure and hybrid GelMA/HAMA scaffolds. The swelling rate was very fast during the first 30–90 min (depending on the hydrogel composition and concentration), followed by a more sustained phase, which became stationary at ca. 10 h for pure HAMA and pure and hybrid hydrogels containing 10% GelMA, and at ca. 25 h for the remaining compositions, respectively. The swelling degree (SD) decreased as the total polymer concentration increased, ranging from 2400 to 6700% and 470 to 1480% for pure HAMA and GelMA hydrogels, respectively, as a consequence of the larger DoM of the latter. The water absorption capacity is attributed to both the hydrophilic moieties present in HA and Gel chains and the own structure of the hydrogel scaffolds, whereas the existing dissolution resistance originates from the crosslinking of the polymeric chains. Hydrophilicity may decrease when the polymers and proteins are cross-linked because the hydrophilic groups (OH, NH_2_, and COOH) are consumed through the crosslinking process.

In the case of hybrid GelMA/HAMA scaffolds, the SD was among that of pure HAMA and GelMA hydrogels and became lower as the GelMA concentration within the scaffold increased. This fact can be related to a decrease in the hydrogel porosity as well as an increase in the crosslinking density, which, in turn, diminished the hydrogel flexibility and expansion capacity [12,64]. In addition, the rate of solvent absorption increased with the HAMA/GelMA ratio. Nevertheless, it must also be considered that SD values can be lower than those obtained when using pure water as solvent, as the relatively high ionic strength of PBS may facilitate the creation of strong hydrogen bonding within the hydrogel matrix, precluding further solvent absorption [65].

On the other hand, experimental SD kinetic data were fitted to Equation (6), which describes the swelling behavior (Table 2). k was directly related to the solvent absorption velocity inside the hydrogel matrix and followed the sequence pure GelMA < hybrid HAMA/GelMA < pure HAMA scaffolds, in agreement with previous data. The presence of HAMA in the hybrid scaffolds even at a low concentration largely increased the *k* values compared with pure GelMA ones [66]. On the other hand, *n* values were lower than 0.5, which agrees with a Fickian diffusion model of the solvent, that is, its diffusion velocity inside pores is slower than the relaxation of polymeric chains [67,68]. Our data corroborate that the highest SD corresponded to scaffolds with relatively high porosity in agreement with SEM and porosity data, offering more space for water storage [69]. If water/aqueous buffer is also absorbed by the hydrogel wall and polymeric chains, scaffolds with large polymeric concentrations should show the highest adsorption capacity, in contrast to the observations reported here.

### 2.7. In Vitro Biodegradation

To evaluate the biodegradability of the prepared hydrogel scaffolds, they were immersed in PBS buffer containing the enzyme collagenase, which is present in the physiological environment of the human body and has the ability to degrade collagen/Gel. Figure 7e shows the extent of degradation of the photo-crosslinked polymeric scaffolds after 24 h in the presence of the enzyme; conversely, no signs of degradation by network erosion or dissolution were noted in the absence of collagenase within the timeframe of experiments. Non-crosslinked Gel and HA scaffolds were dissolved in just 6 h independently of the presence of the enzyme (data not shown).

Pure GelMA scaffolds were completely degraded after 24 h of incubation in the presence of collagenase. Such fast degradation might be motivated by the high activity of the enzyme after being changed each 4 h. Conversely, HAMA hydrogels were hardly affected by the enzyme, as expected. GelMA/HAMA hybrid hydrogels showed a degradation pattern in the presence of collagenase that was dependent on the composition, that is, the larger the content in GelMA, the larger the scaffold weight loss. Moreover, a larger HAMA proportion in the scaffold´s composition attenuated the enzymatic degradation. This behavior may be attributed to the formation of stronger covalent bonds between HAMA and GelMA after photopolymerization, making hydrogels harder to break after an enzymatic attack. Then, the presence of HAMA seems to improve the stability of the polymeric network, and the degradation pathway can be controlled by the HAMA/GelMA ratio in order to achieve the required conditions for a biomimetic ECM for a prospective TE application.

In summary, given the excellent biocompatibility of GelMA and HAMA (if remaining impurities from the photopolymerization process are eliminated through suitable washing steps) and the easiness to tune scaffold structure and physical properties, the present hybrid GelMA/HAMA hydrogels are envisaged as potential materials to be used either as injectable (if not or only lightly crosslinked and/or at low concentrations) or implantable scaffolds for tissue engineering applications, overcoming some of the drawbacks of their pure counterparts, such as the lack of adhesiveness of HAMA or the weak mechanical strength of GelMA ones, respectively. The present hybrid GelMA/HAMA hydrogels showed a structure of well-intermixed of polymeric chains belonging to both HAMA and GelMA, in which, besides chemical crosslinkings, physical bonding (mainly electrostatic interactions, but hydrogen bonding and/or hydrophobic interactions may also have an influence) between polymer chains seems to play an active role in providing an additional thermal and mechanical stability to the hybrid scaffolds compared with their pure counterparts. In this respect, the hybrid scaffolds are able to reproduce the mechanical and structural properties of different tissues by simply changing the polymer composition and/or concentration, providing hydrogel scaffolds with elastic moduli ranging from some few Pa up to several dozens of MPa, more thermally stable than their pure counterparts, and inner structures with porosities larger than 70%, which should ensure a correct permeation of nutrients and facilitate cell colonization and growth. In addition, all hybrid hydrogels showed an excellent swelling ability, which decreases as the GelMA concentration in the formulation increases (and the total polymer mass), and good resistance to enzymatic degradation, which is enhanced as more GelMA is added in the scaffold structure.

## 3. Materials and Methods

### 3.1. Materials

Gelatin type B (from bovine skin), phosphate buffered saline (1X, pH 7.4), methacrylic anhydride (MA), 2-hydroxy-1-[4-(hydroxyethoxy)phenyl]-2-methyl-1-propanone (Irgacure 2959), collagenase, sodium hydroxide, and deuterium oxide were purchased from Sigma-Aldrich. Hyaluronic acid sodium salt (HA, MW = 80,000–100,000 kDa) was obtained from Carbosynth. Dialysis Tubing Spectra/Por 4 (12-14 kD MWCO) was purchased from Spectrum Lab Inc. Teflon molds of 15 mm diameter and 40 mm of height were fabricated at a local company. Other reagents were of the highest purity available. MilliQ water was used in all experiments.

### 3.2. Gelatin and Hyaluronic Acid Methacrylation

Methacrylation of Gel and HA was performed following previously reported protocols [33,34]. For Gel, 1.1 mL of MA at 1% (*v/v*) was added dropwise to 100 mL of a 10% *w/v* gelatin solution in PBS, pH 7.4, at 50 °C, under magnetic stirring for 3 h. The reaction was stopped with the addition of 400 mL of PBS and dialyzed for 7 days using a 12–14 kDa membrane in deionized water at 40 °C to remove excess reagents. Methacrylation of HA was performed by adding dropwise 1.1 mL of MA at 1% (*v/v*) to 100 mL of a 1% (*w/v*) HA solution in PBS buffer, pH 7.4, at 4 °C, under magnetic stirring for 24 h. The pH of the solution was kept between 8 and 10 with the addition of 5 M NaOH, until no further pH changes were detected, which indicated the reaction was complete. The solution was dialyzed for 4 days with a 12–14 kDa membrane in deionized water at 4 °C. GelMA and HAMA were frozen in liquid nitrogen and lyophilized, and the obtained powder material was stored at −20 °C until further use. The degree of methacrylation (DoM) of GelMA and HAMA was evaluated by proton nuclear magnetic resonance (^1^H NMR). ^1^H NMR spectra of 30 mg/mL of Gel, GelMA, HA, and HAMA were acquired using a Bruker DRX 500 (Bruker, Billerica, MA, USA) 400 MHz spectrometer in deuterium oxide. The DoM of GelMA was calculated by normalizing the spectra against the phenylalanine signal at 7.1–7.6 ppm for five protons and integrating the methacrylate protons at ca. 5.4–6.0 ppm. The DoM was calculated using a phenylalanine content of 0.126 mmol/g [70], as follows:

(1) $$DoM=\frac{Am\left(methacryloyl\;groups\right)\;}{Ap\left(phenylalanine\right)}\times 5H\times phenylalanine\;content\left(\frac{mmol}{g}\right)$$

where A_m_ is the area of the 5.4–6.0 ppm methacryl peak and A_p_ is the area of the 7.1–7.6 ppm methacryl protons. For HAMA, the DoM was obtained using the ratio of the area of methacrylate protons (5.6–6.2 ppm) with respect to the HA methyl protons (~2.0 ppm) [25,71,72] using the following equation:

(2) $$DoM=100\%\times \left(\frac{metacrylated\;protons}{methyl\;protons\;of\;HA\;}\right)$$

### 3.3. Hydrogel Preparation

Pure and hybrid hydrogels were prepared at different GelMA (2, 6, 10% *w/v*) and HAMA (1, 3, 5% *w/v*) concentrations (Table 1). Briefly, hydrogels of pure GelMA were produced by mixing the polymer precursor in PBS at 37 °C with 0.5% (*w/v*) of Irgacure^®^ 2959 photoinitiator. Pure HAMA and hybrid GelMA/HAMA hydrogels were obtained by mixing different polymer concentrations with 0.1% (*w/v*) of the photoinitiator. The polymeric mixtures were poured in fabricated PTFE molds (15 mm diameter, 40 mm height) and cured with UV light at 365 nm (4 W/cm^2^) for 10 min. The unreacted polymers were rinsed with PBS.

### 3.4. Gel Fraction

The gel percent of the hydrogels was determined gravimetrically. The different lyophilized hydrogels were previously weighted, and subsequently immersed in 10 mL of PBS for 96 h at room temperature under slight shaking, and then freeze-dried. The gel percent was calculated by the following equation:

(3) $$Gel\;\left(\%\right)=100\times \frac{W_{w}}{W_{i}}$$

where w_i_ and w_w_ are the weights (g) of the hydrogel before and after washing to extract the soluble parts, respectively. All the experiments were performed in triplicate.

### 3.5. Apparent Density 

Hydrogel apparent density, *ρ*, was evaluated from the ratio of the lyophilized hydrogel weight to volume through the following equation:

(4) $$\rho =\frac{w}{\pi \times {\left(D/2\right)}^{2}\times H}$$

where ρ is the apparent density (g/cm^3^), w is the weight of the hydrogel (g), D the diameter (cm), and H the thickness of the hydrogel (cm), respectively.

### 3.6. Fourier-Transform Infrared Spectroscopy 

The chemical characterization of the hydrogel was done by attenuated-reflectance Fourier-transform infrared spectroscopy (ATR-FTIR). Dried hydrogels were slightly humidified and placed on a microsample cup. Data acquisition was performed using a FTIR spectrometer (Varian 670, Agilent, Santa Clara, CA, USA) coupled to a mapping microscope (Varian 620-IR, Agilent, Santa Clara, CA, USA) and an ATR diamond accessory. The samples were analyzed in the interval from 400 to 4000 cm^−1^ with a spectral resolution of 4 cm^−1^ and 64 scans min^−1^ for a total of 100 scans for each spectrum.

### 3.7. Raman Imaging

Raman spectroscopy data of pure (controls) and hybrid hydrogels were obtained with a WITec Confocal Raman microscope Alpha 300R+ (Ulm, Germany). Dried hydrogel samples were homogenized with PBS to avoid potential background fluorescence. Surface and in-depth distribution of the two different polymers inside hybrid hydrogels were determined using a frequency doubled laser at 532 nm at an output power of 7 mW and 600 mm grating. Raman spectra for image compositions were recorded using a 100X Zeiss, EC Epiplan-Neofluar DIC objective (Oberkochen, Germany) with a numeric aperture of 0.9. Image resolution was set at 1024 × 127 active pixels, with a total of 22,500 spectra per image at a scan speed of 20 s per line and an integration time per pixel of 0.13 s. Data acquisition was driven by the WITec Control software (Ulm, Germany). Peak identification in hybrid and pure hydrogels was recorded and compared.

### 3.8. Thermal Analysis

The thermal behavior of the hydrogels was analyzed by thermo-gravimetrical analysis (TGA) and differential scanning calorimetry (DSC). Their decomposition behavior and thermal stability were analyzed using a TGA 55 thermogravimeter analyzer (TA Instruments, New Castle, DE, USA). Then, 10 mg of hydrogel samples were heated in a platinum pan from 30 to 900 °C at 50 °C/min under N_2_ atmosphere. DSC data were acquired using a Q-100 differential scanning microcalorimeter (TA Instruments, USA), for which 3 to 5 mg of lyophilized hydrogel was placed into hermetic aluminum pans and heated from −40 to 300 °C at a heating ramp of 10 °C/min under N_2_ atmosphere.

### 3.9. Oscillatory Rheological Measurements

The photo-crosslinking kinetics of hydrogel formation was studied using an Anton-Paar MCR301 constant stress rheometer with a 15 mm parallel-plate geometry (lower glass plate) and a gap of 240 μm. The polymerization was carried out for methacrylated gelatin (GelMA, 2, 6, and 10% wt.), methacrylated hyaluronic acid (HAMA, 1, 3, and 5% wt.), and mixtures of GelMA-HAMA with Irgacure 2959 photoinitiator (0.1% wt. for HAMA and hybrid HAMA/GelMA and 0.5% wt. for GelMA) and irradiated for 10 min with UV light, starting at 50 s of the oscillatory time sweeps experiments (365 nm, UV Omnicure S1500 at 30% of its nominal capacity of 23 W/cm^2^).

The mechanical properties were determined for cross-linked hydrogels using the aforementioned rheometer. Firstly, oscillatory strain sweep measurements were performed in order to obtain the linear viscoelastic region (LVR) using a deformation range from 0.1 to 100%, at a frequency (ω) of 6.28 rad/s (1 Hz), at 37 °C. The LVR is defined as the deformation range where the storage modulus (G′) is independent of the applied deformation (%γ). After a critic deformation ($\%\gamma_{c}$), G′ exhibits a change in its slope, a decrease as the deformation increases, due to a rupture in the hydrogel microstructure [17]. Once the LVR was determined, changes in the mechanical properties as a function time were monitored for a second set of cross-linked hydrogels with oscillatory time sweeps experiments from 0.1 to 100 rad/s, at a deformation value within the LVR, at 37 °C. All samples were initially stabilized for 3 min, while a humidification chamber unit was placed around the geometries to avoid water evaporation.

### 3.10. Mechanical Compression Tests

Pure HAMA, GelMa, and hybrid scaffolds were subjected to unidirectional compression tests in a tensile bench with a 30 kg load cell (TA.TX*Plus*, Stable Micro Systems, Ltd., Godalming, UK) at a crosshead speed of 1 mm/min. All the experiments were performed at room temperature (25 °C), atmospheric pressure, and 45% relative humidity. Young’s modulus (E) was calculated from the slope in the linear section of the stress−strain curve at low strains (<20%). Three replicates were used for each hydrogel composition.

### 3.11. Field-Emission Scanning Electron Microscopy

The morphology and microstructure of the hydrogels were evaluated using field-emission scanning electron microscopy (FESEM, Zeiss Ultra-Plus, Potsdam, Germany). Hydrogels were first swollen for two days in PBS at 37 °C, and then frozen in liquid nitrogen. Next, the samples were fixed with conductive adhesive on aluminum supports, sputter-coated with iridium, and observed at an accelerating voltage of 20 kV. The average cross section area of the pores was calculated using ImageJ software (National Institute of Health, Bethesda, MD, USA).

### 3.12. Mercury Immersion Porosimetry

Textural properties of the scaffolds (total pore volume (V_p_), pore size distribution, and open porosity (ε_MIP_)) before and after storage were measured through mercury intrusion porosimetry (MIP) with an Autopore IV equipment (9500 model, Micromeritics, Norcross, GA, USA). MIP was operated with a 3 mL penetrometer for solids and at working pressures ranging from 0.07 to 1724 bar. Porosity and mean pore size (MIP—mean pore size) were determined using the Washburn equation from the variation of the intruded volume of mercury (V_p,MIP_) in the scaffolds with the increase of pressure [73].

### 3.13. Swelling

The swelling behavior of hydrogels was measured gravimetrically. Pure dried GelMA, HAMA, and hybrid hydrogels were submerged in 10 mL of PBS buffer, pH 7.4, at 37 °C. The weight change was initially recorded every 60 min for 12 h, and then every 12 h until the equilibrium swelling was reached. The wet weight of the swollen hydrogels was then determined after gently removing the excess liquid using kimwipes. All the experiments were performed in triplicate. The swelling degree (in percentage) was calculated as follows:

(5) $$Swelling\;degree\;\left(SD\right)=\frac{wt.\;of\;wet\;sample-wt.\;of\;dried\;sample}{wt.\;of\;dried\;sample}\times 100$$

Swelling kinetic data were fitted to the following equation:

(6) $$\frac{SD_{t}}{SD_{eq}}=kt^{n}$$

where *SD_t_* is the swelling degree at time *t*; *SD_eq_* is the swelling degree at equilibrium; *n* is the diffusional diffusion of the solvent; and *k* is the constant, which changes according to the polymeric hydrogel structure.

### 3.14. In Vitro Degradation 

Pure and hybrid hydrogels were sterilized and neutralized in alcohol overnight, weighted, washed with PBS buffer at 37 °C, and then rehydrated with an excess of the same buffer for 24 h prior to the degradation assay. The enzymatic degradation was carried out at 37 °C in 15 mL of PBS (pH 7.4) with 3.0 U/mL of collagenase type II. The enzyme solution was refreshed each 4 h to ensure a continuous enzymatic activity. The hydrogels were removed each 2 h out from the medium, washed with distilled water, and freeze-dried to determine the dry weight of the remaining polymer. Each experiment was conducted in triplicate and the average value was taken as the percentage of degradation. The degradability ratio D was calculated as follows:

(7) $$Degradability\;\left(D\%\right)=\frac{wt.\;of\;sample\;before\;degradation\;test-wt.\;of\;sample\;at\;specific\;day}{wt.\;of\;sample\;before\;degradation\;test}\times 100$$

## 4. Conclusions

Pure and hybrid HAMA/GelMA hydrogels were prepared by photochemical crosslinking to produce scaffolds with a suitable structure, porosity, and stiffness for tissue engineering purposes. The scaffolds were structurally and physically characterized by scanning electron microscopy, mercury intrusion porosimetry, attenuated total reflectance Fourier transform infrared and Raman spectroscopies, thermal gravimetric analysis, differential scanning calorimetry, and rheology and compression experiments, and their stability was evaluated by swelling degree and enzymatic degradation tests. The produced hybrid HAMA/GelMA scaffolds have structures and porosities lying in between those of their pure counterparts, being more porous, but disorganized hydrogels with very large swelling degrees at fixed HAMA concentration in the composition, if compared with the pure GelMA counterparts. Conversely, the progressive predominance of GelMA in the scaffolds composition leads to stiffer, more structured, but less porous scaffolds, as also noted when the total polymeric content of the scaffold increases. In addition, the presence of HAMA in the hybrid hydrogels improved the resistance to collagenase degradation thanks to the formation of stronger covalent bonds between HAMA and GelMA after photopolymerization, making scaffolds harder to break after enzymatic attack when compared with pure GelMA hydrogels of a similar composition. In summary, the scaffolds present properties such as tunable porosity, degree of swelling, and stability that make them suitable as supporting biomaterials in different tissue regeneration and repair applications.

## Figures and Tables

**Table 1:** Summary of compositions and physico-chemical properties of scaffolds.

Samples	Gel Fraction (%)	E (kPa)	Density (g/mL)	ε_MIP_ (%)	MIP—Mean Pore Diameter (μm)
1% HAMA	100 ± 1	1.0 ± 0.2	0.065 ± 0.004	94 ± 2	89 ± 12
3% HAMA	100 ± 1	6.6 ± 0.9	0.072 ± 0.006	75 ± 3	54 ± 10
5% HAMA	100 ± 1	12.4 ± 2.1	0.084 ± 0.006	65 ± 1	32 ± 6
2% GelMA	70 ± 6	0.8 ± 0.1	0.072 ± 0.005	89 ± 2	26 ± 4
6% GelMA	79 ± 3	2.1 ± 0.3	0.105 ± 0.006	77 ± 3	17 ± 3
10% GelMA	79 ± 1	3.1 ± 0.5	0.157 ± 0.008	67 ± 4	11 ± 2
2% GelMA-1% HAMA	71 ± 5	2.0 ± 0.2	0.070 ± 0.002	91 ± 1	31 ± 4
2% GelMA-5% HAMA	80 ± 2	12.8 ± 1.9	0.079 ± 0.003	76 ± 2	19 ± 3
6% GelMA-1% HAMA	83 ± 3	5.6 ± 1.1	0.101 ± 0.006	81± 4	22 ± 5
6% GelMA-5% HAMA	87 ± 2	9.0 ± 1.2	0.103 ± 0.004	74 ± 4	20 ± 2
10% GelMA-1% HAMA	91 ± 6	9.5 ± 1.7	0.149 ± 0.009	68 ± 5	13 ± 3
10% GelMA-5% HAMA	95 ± 4	18.3 ± 2.4	0.192 ± 0.013	54 ± 6	8 ± 2

**Table 2:** Swelling kinetic fitting data of pure and hybrid scaffolds according to Equation (6).

Scaffolds	k	n
1% HAMA	0.80 ± 0.09	0.22 ± 0.07
3% HAMA	0.74 ± 0.06	0.23 ± 0.05
5% HAMA	0.72 ± 0.03	0.29 ± 0.02
2% GelMA	0.70 ± 0.07	0.28 ± 0.05
6% GelMA	0.65 ± 0.05	0.30 ± 0.04
10% GelMA	0.51 ± 0.01	0.31 ± 0.01
2% GelMA-1% HAMA	0.69 ± 0.11	0.29 ± 0.08
2% GelMA-5% HAMA	0.62 ± 0.07	0.35 ± 0.06
6% GelMA-1% HAMA	0.70 ± 0.10	0.25 ± 0.08
6% GelMA-5% HAMA	0.66 ± 0.04	0.29 ± 0.05
10% GelMA-1% HAMA	0.77 ± 0.08	0.25 ± 0.09
10% GelMA-5% HAMA	0.67 ± 0.02	0.35 ± 0.02

## Data Availability

The data that supports the findings of this study are available from the corresponding authors, J.F.A. and P.T., upon reasonable request.

## Appendix A

**Table A1:** Gelation time (t_gel_) for HAMA, GelMA, and hybrid HAMA/GelMA hydrogels obtained from the onset of the gelation process.

Sample	Concentration (*w*/*v*%)	$t_{gel}\;(\mathrm{Min})$
HAMA	1	1.92
	3	1.00
	5	0.88
GelMA	2	0.94
	6	0.52
	10	0.35
1% HAMA/X% GelMA	2	1.69
	6	0.93
	10	1.00
5% HAMA/X% GelMA	2	0.91
	6	0.86
	10	0.77
